# Health Care Access Dimensions and Racial Disparities in End-of-Life Care Quality among Patients with Ovarian Cancer

**DOI:** 10.1158/2767-9764.CRC-23-0283

**Published:** 2024-03-18

**Authors:** Shama Karanth, Oyomoare L. Osazuwa-Peters, Lauren E. Wilson, Rebecca A. Previs, Fariha Rahman, Bin Huang, Maria Pisu, Margaret Liang, Kevin C. Ward, Maria J. Schymura, Andrew Berchuck, Tomi F. Akinyemiju

**Affiliations:** 1 UF Health Cancer Center, University of Florida, Gainesville, Florida.; 2 Department of Population Health Sciences, Duke University School of Medicine, Durham, North Carolina.; 3 Division of Gynecologic Oncology, Duke Cancer Institute, Duke University School of Medicine, Durham, North Carolina.; 4 Duke Cancer Institute, Duke University School of Medicine, Durham, North Carolina.; 5 Department of Biostatistics and Kentucky Cancer Registry, Univ of Kentucky, Lexington, Kentucky.; 6 Division of Preventive Medicine and O'Neal Comprehensive Cancer Center, University of Alabama at Birmingham, Birmingham, Alabama.; 7 Division of Gynecologic Oncology, Department of Obstetrics & Gynecology, and O'Neal Comprehensive Cancer Center, University of Alabama at Birmingham, Birmingham, Alabama.; 8 Georgia Cancer Registry, Emory University, Atlanta, Georgia.; 9 New York State Cancer Registry, New York State Department of Health, Albany, New York.

## Abstract

This study investigated the association between health care access (HCA) dimensions and racial disparities in end-of-life (EOL) care quality among non-Hispanic Black (NHB), non-Hispanic White (NHW), and Hispanic patients with ovarian cancer. This retrospective cohort study used the Surveillance, Epidemiology, and End Results–linked Medicare data for women diagnosed with ovarian cancer from 2008 to 2015, ages 65 years and older. Health care affordability, accessibility, and availability measures were assessed at the census tract or regional levels, and associations between these measures and quality of EOL care were examined using multivariable-adjusted regression models, as appropriate. The final sample included 4,646 women [mean age (SD), 77.5 (7.0) years]; 87.4% NHW, 6.9% NHB, and 5.7% Hispanic. In the multivariable-adjusted models, affordability was associated with a decreased risk of intensive care unit stay [adjusted relative risk (aRR) 0.90, 95% confidence interval (CI): 0.83–0.98] and in-hospital death (aRR 0.91, 95% CI: 0.84–0.98). After adjustment for HCA dimensions, NHB patients had lower-quality EOL care compared with NHW patients, defined as: increased risk of hospitalization in the last 30 days of life (aRR 1.16, 95% CI: 1.03–1.30), no hospice care (aRR 1.23, 95% CI: 1.04–1.44), in-hospital death (aRR 1.27, 95% CI: 1.03–1.57), and higher counts of poor-quality EOL care outcomes (count ratio:1.19, 95% CI: 1.04–1.36). HCA dimensions were strong predictors of EOL care quality; however, racial disparities persisted, suggesting that additional drivers of these disparities remain to be identified.

**Significance:**

Among patients with ovarian cancer, Black patients had lower-quality EOL care, even after adjusting for three structural barriers to HCA, namely affordability, availability, and accessibility. This suggests an important need to investigate the roles of yet unexplored barriers to HCA such as accommodation and acceptability, as drivers of poor-quality EOL care among Black patients with ovarian cancer.

## Introduction

End-of-life (EOL) care refers to health care for terminally ill patients, which is patient- and family-centered, and focused on improving the quality of a patient's remaining life while ensuring a “good” death, whereby the death experience is personalized to match the patient's preference (1, 2). High-quality EOL care avoids treatment that does not confer any physiologic benefits, thus reducing aggressive medical interventions, minimizing hospitalizations and utilization of the intensive care unit (ICU), and ensuring the patient dies at a preferred place, usually not at the hospital (3). The benefits of high-quality EOL care include improved quality of life, better symptom management, reduced anxiety and depression, decreased caregiver distress, and drastically lower health care cost (4–6).

Despite known benefits of EOL care, many patients do not receive this approach (1, 3) with only 51.6% of Medicare decedents receiving hospice services in 2019 (7), and nearly one-third of Medicare patients receiving care in the ICU during the last month of life (8). Moreover, quality EOL care is characterized by marked racial disparities at regional (9–11), and national levels (12–14). Such disparities appear to be pervasive among patients with ovarian cancer (9, 12, 13, 15), for whom EOL care is highly relevant, with poor 5-year survival rates of 36% and 46% for non-Hispanic Black (NHB) and non-Hispanic White (NHW) women, respectively (16). NHB patients with ovarian cancer are less likely to receive hospice care, more likely to receive chemotherapy, and more likely to receive two or more hospitalizations and ICU admissions in the last 30 days of life compared with NHW patients (9, 12, 13, 17). Similarly, Hispanic patients with ovarian cancer are less likely to enroll and die in hospice and more likely to be admitted into ICU, compared with NHW patients (9).

While trends and disparities in EOL care among patients with ovarian cancer are well documented and mirror disparities in access to palliative and hospice use in general (10, 12, 15, 18), the role of key health care access (HCA) dimensions in quality EOL care has not been well characterized. Findings are critical for informing interventions or recommendations aimed at improving access among patients with ovarian cancer (3).

HCA, a multidimensional concept that captures the degree of fit between a patient and the health care system, offers a useful framework for studying disparities in quality EOL care among patients with ovarian cancer (3, 19–21). HCA is composed of five distinct and interrelated dimensions of access to care, namely, availability (type, quality, and quantity of health care resources), affordability (ability to pay for health care), accessibility (proximity of health care resources to a patient), accommodation (organization of health care resources to patient's constraints and preferences), and acceptability (patient's attitude, perceptions, and quality of interactions with health care providers; ref. 20). Prior research has demonstrated the utility of examining multiple HCA dimensions for understanding barriers and challenges to equitable treatment quality (3, 21). Therefore, this study aims to investigate the association of three HCA domains estimable from the Surveillance, Epidemiology, and End Results (SEER)-Medicare database (availability, accessibility, and affordability) in relation to racial disparities in EOL care quality among NHB, NHW, and Hispanic patients with ovarian cancer.

## Materials and Methods

### Study Population

**FIGURE 1:**
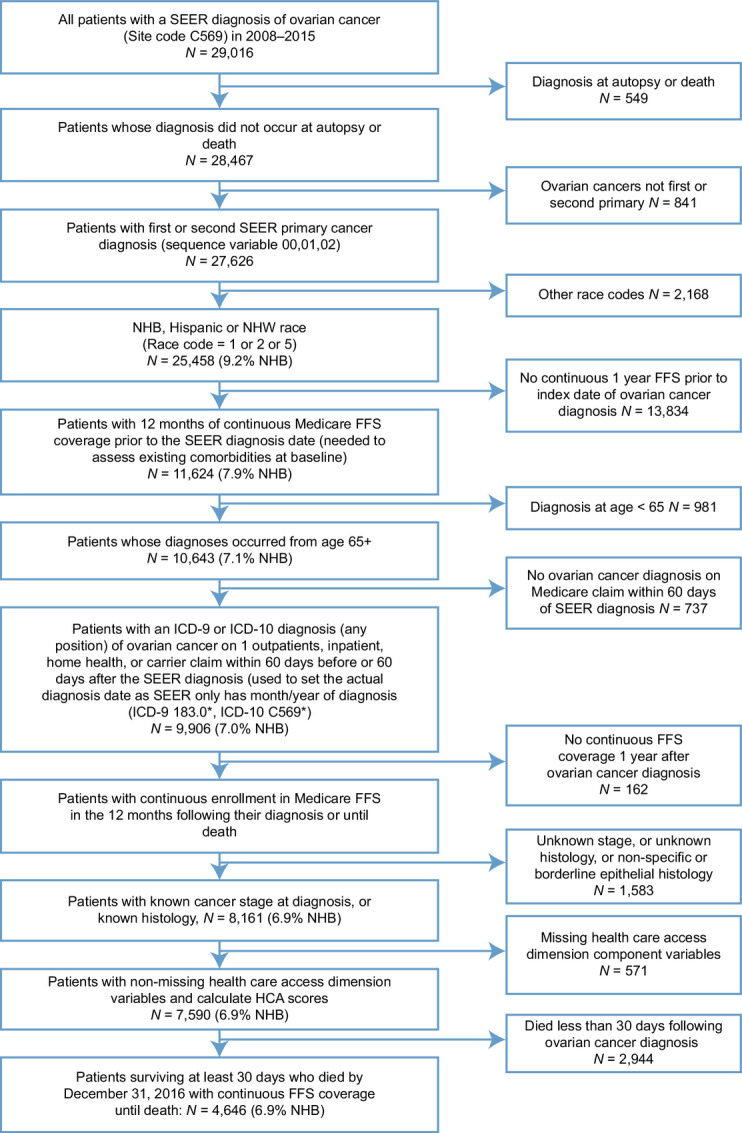
Participant flowchart for NHB, Hispanic and NHW patients with ovarian cancer, SEER-Medicare 2008–2015. Percentage of NHB patients presented for sensitivity purposes.

The study cohort included NHB, NHW, and Hispanic women ages ≥65 years, diagnosed between 2008 and 2015 with first or second primary ovarian cancer of any histologic type (C569) in the SEER-Medicare linked dataset (22). These women had at least 12 months of continuous enrollment in Medicare fee-for-service (FFS) parts A and B prior to diagnosis and continuous FFS coverage from diagnosis until death. They also survived for at least 30 days following their initial ovarian cancer diagnosis and died by December 31, 2016. These patients also were eligible for the National Comprehensive Cancer Network (NCCN) guideline recommendations for systemic therapy based on their cancer stage, grade, and histology at diagnosis (excluding patients with borderline epithelial or nonspecific histology, epithelial histology with stage <IC and grade <3, stage I dysgerminoma histology, stage I malignant sex cord-stromal histology, or stage I and grade I immature teratoma histology). We followed histology categorizations as defined in Koshiyama and colleagues 2014 (23). The requirement that patients had a stage/histology that was eligible for systemic therapy based on NCCN guidelines was important because one of the outcomes considered was aggressive use of systemic therapy during EOL. The study cohort selection process is detailed in Fig. 1. This study was approved by the Institutional Review Board of Duke University (Durham, NC).

### EOL Care Quality Metrics

Using linked Medicare claims, we assessed established measures of intensive EOL cancer care (24), including receipt of systemic therapy in the last 14 days of life, initiation of a new systemic therapy within the last 30 days of life, hospital admission in the last 30 days of life, ICU admission within the last 30 days of life, in-hospital death, two or more emergency room (ER) visits in the last 30 days of life, no hospice initiation prior to death, and late hospice initiation within the last 3 days of life (25). These Current Procedural Terminology codes were used to assess receipt of systemic therapies: Q0083-Q0085, G0355-G0363, J8501-J9999, 96400-96549. Hospital admission and hospice initiation were identified by the presence of inpatient and hospice claims, respectively. ICU stays were identified by the presence of an inpatient claim with revenue center codes 0200-0209; ER visits were identified by the presence of outpatient or inpatient claims with revenue center codes 0450-0459 or 0981, or inpatient claims with ER charges. Hospital death was identified by the presence of an inpatient claim with a discharge status of “died.” Counts of poor-quality EOL outcomes were created by summing the individual poor-quality EOL outcomes for each patient.

### SEER Patient Demographics and Clinical Characteristics

We obtained data on patient and clinical characteristics from the SEER database including race and ethnicity, age at diagnosis, sex, cancer stage at diagnosis, histology at diagnosis (26), marital status, geographic region of residence, and residence in a metropolitan area. We used validated coding algorithms to assess patient comorbidities and calculate Charlson comorbidity index scores 12 months prior to ovarian cancer diagnosis (27, 28).

### HCA Dimension Scores

A total of 35 patient, census tract, and regional level variables (Supplementary Table S1) measuring dimensions of health care affordability (i.e., census tract poverty rates and educational attainment), availability (i.e., number of hospitals and specialists available per capita in the patient's county or health care referral region of residence), and accessibility (i.e., residence in metropolitan versus rural area, distance traveled to care based on patient and care facility zip codes) were assessed. The study measures were sourced from the SEER-Medicare database, Area Healthcare Resource File, and Dartmouth Atlas of Healthcare; the full details of the methods are provided in the Supplementary Materials and Methods. Confirmatory factor analysis (CFA) was used to identify the most influential variables for each aspect of HCA and to create composite scores of affordability, availability, and accessibility for each patient, which ranged from −3 to 4 (Supplementary Table S2). Lower availability scores correspond with fewer numbers of physicians and hospitals in the patient's region of residence and lower average hospital quality; lower accessibility scores indicate greater travel distance to health care facilities or residence in rural areas; lower affordability scores indicate residence in neighborhoods with higher poverty rates or lower educational attainment. Full description of the methods used to create HCA dimension scores are available in Gupta, Chen, Wilson, Huang, Pisu, Liang, Previs, Moss, Ward, Schymura, Berchuck, and Akinyemiju (29). In addition, the CFA for the HCA domains is described in the Supplementary Materials and Methods.

### Statistical Methods

Descriptive analyses using *χ*^2^ tests, Wilcoxon rank-sum tests, and Kruskal–Wallis tests were performed to examine the bivariate associations between patient race and ethnicity, patient characteristics, and HCA factor scores. EOL care quality metrics were reported as frequencies and percentages, stratified by race and ethnicity. Group differences were tested using *χ*^2^ tests and the Cochran—Mantel–Haenszel test for nonzero correlation.

To assess the associations between patient race and ethnicity, HCA scores, and each EOL care quality metric, we used multivariable-adjusted log-binomial regression to estimate the relative risk for outcomes with >10% prevalence (i.e., hospital admission in the last 30 days of life, ICU admission within the last 30 days of life, in-hospital death, and no hospice initiation prior to death). For rare outcomes (<10%; i.e., receipt of systemic therapy in the last 14 days of life, initiation of a new systemic therapy within the last 30 days of life, two or more ER visits in the last 30 days of life, no hospice initiation prior to death, and late hospice initiation within the last 3 days of life), we used logistic regression to calculate odds ratios (OR) instead. Associations between patient race and ethnicity, HCA scores, and counts of poor EOL care quality metrics were modeled using multivariable-adjusted negative binomial regression to model count ratios. All models were minimally adjusted for patient characteristics, including age at diagnosis, cancer stage at diagnosis, tumor histology, geographic region of residence, year of diagnosis, and comorbid conditions at cancer diagnosis, and one HCA score. The fully-adjusted model included patient characteristics, and all three HCA scores as covariates. We included interaction terms between race and ethnicity and HCA scores in the fully-adjusted model to examine whether the associations between HCA scores and outcomes were moderated by race and ethnicity. Variable collinearity was assessed using the variance inflation factor method. No adjustment variables were excluded on the basis of a variance inflation factor threshold of 2.5.

Finally, we performed three sets of sensitivity analyses to examine the robustness of our findings given three potential sources of confounding: (i) whether associations differed or remained consistent for patients who died from ovarian cancer, (ii) whether findings remained consistent when including patients without a known stage or histology eligible for systemic therapy, and (iii) whether survival time (i.e., difference in days between death date and ovarian cancer diagnosis date) confounded associations between EOL care quality and HCA dimensions. To examine robustness of our findings to death from ovarian cancer as a potential confounder, we ran fully-adjusted models on data for the subset of patients whose cause of death was ovarian cancer while excluding patients who died from other causes. For the second potential confounder, we relaxed the requirement for known stage/histology eligible for systemic therapy following NCCN guidelines, thus including patients that did not satisfy this requirement. Finally, for the third potential confounder, we statistically adjusted for survival time, defined as the difference between date of death and date of ovarian cancer diagnosis.

### Data Availability

The data analyzed in this study are available from SEER-Medicare, which is administrated by the NCI. Restrictions apply to the use of these data, which was used under license by the authors. Access to the data is controlled, and can be requested for research purposes at the SEER-Medicare website (https://healthcaredelivery.cancer.gov/seermedicare/obtain/).

### Ethics Statement

This study was approved by the Institutional Review Board of Duke University (Pro#00101872).

## Results

### Study Population Description

The study's characteristics and descriptive statistics are shown in Table 1. A total of 4,646 patients with ovarian cancer died during the study follow-up and met all the inclusion criteria: 4,061 (87.4%) NHW patients, 322 (6.9%) NHB patients, and 263 (5.7%) Hispanic patients. On the basis of the cause of death information, 3,195 (68.8%) patients died of ovarian cancer, and the majority (*n* = 4,176, 89.9%) were diagnosed with stage III or IV disease. Type II epithelial histology was most common in each subgroup [NHW: 2,907 (71.5%); NHB: 228 (70.8%); Hispanic: 183 (69.6%)], with a high frequency of serous high grade (36.12%), undifferentiated epithelial (28.76%), and serous low/unknown grade (21.44%) subtypes overall in the study cohort. NHB patients had a slightly higher comorbidity burden on average than NHW and Hispanic patients (*P <* 0.001). On average, compared with NHW patients, NHB and Hispanic patients had lower affordability scores and higher accessibility scores (*P* < 0.001), whereas availability scores did not differ by race (*P* = 0.121; Table 1).

**TABLE 1:** Baseline characteristics of patients with ovarian cancer who died during study follow-up stratified by race and ethnicity (*N* = 4,646)

Variable	NHW	NHB	Hispanic	*P*-value
*N*	4,061	322	263	
Patient characteristics at diagnosis				
Age at ovarian cancer diagnosis, mean (SD)	77.7 (7.0)	76.3 (6.6)	76.8 (6.9)	<0.001
Cause of death was ovarian cancer	2,751 (67.7%)	251 (78.0%)	193 (73.4%)	<0.001
Tumor stage at dx				0.142
I	177 (4.4%)	14 (4.3%)	12 (4.6%)	
II	232 (5.7%)	21 (6.5%)	14 (5.3%)	
III	1,926 (47.4%)	126 (39.1%)	116 (44.1%)	
IV	1,726 (42.5%)	161 (50.0%)	121 (46.0%)	
Histology				0.31
Type I epithelial	1,143 (28.1%)	>85	80 (30.4%)	
Type II epithelial	2,905 (71.5%)	228 (70.8%)	183 (69.6%)	
Other	13 (0.3%)	<11	0 (0.0%)	
Geographic region				<0.001
Midwest	516 (12.7%)	<50	<11	
Other/NA	360 (8.9%)	<50	<11	
Northeast	882 (21.7%)	76 (23.6%)	43 (16.3%)	
South	611 (15.0%)	98 (30.4%)	11 (4.2%)	
West	1,692 (41.7%)	64 (19.9%)	195 (74.1%)	
Median comorbidity score, IQR, median (Q1, Q3)	2.0 (1.0, 4.0)	3.0 (2.0, 5.0)	2.0 (1.0, 5.0)	<0.001
Myocardial infarction	112 (2.8%)	<15	<11	0.360
Hypertension	2,649 (65.2%)	276 (85.7%)	175 (66.5%)	<0.001
Peripheral vascular disease	367 (9.0%)	43 (13.4%)	30 (11.4%)	0.021
Congestive heart failure	367 (9.0%)	49 (15.2%)	34 (12.9%)	<0.001
Dementia	64 (1.6%)	<11	<11	0.039
Chronic obstructive pulmonary disease	634 (15.6%)	54 (16.8%)	38 (14.4%)	0.738
Cerebrovascular disease	352 (8.7%)	28 (8.7%)	24 (9.1%)	0.971
Rheumatologic disease	151 (3.7%)	11 (3.4%)	16 (6.1%)	0.140
Peptic ulcer disease	56 (1.4%)	<11	<11	0.958
Mild liver disease	154 (3.8%)	16 (5.0%)	24 (9.1%)	<0.001
End-stage renal disease	265 (6.5%)	41 (12.7%)	25 (9.5%)	<0.001
Diabetes	808 (19.9%)	115 (35.7%)	99 (37.6%)	<0.001
Diabetes with complications	180 (4.4%)	35 (10.9%)	35 (13.3%)	<0.001
Hemiplegia or paraplegia	16 (0.4%)	<11	<11	0.030
Year of diagnosis				0.210
2008	687 (16.9%)	52 (16.1%)	45 (17.1%)	
2009	646 (15.9%)	47 (14.6%)	34 (12.9%)	
2010	552 (13.6%)	39 (12.1%)	35 (13.3%)	
2011	468 (11.5%)	57 (17.7%)	28 (10.6%)	
2012	469 (11.5%)	45 (14.0%)	33 (12.5%)	
2013	483 (11.9%)	35 (10.9%)	35 (13.3%)	
2014	415 (10.2%)	27 (8.4%)	31 (11.8%)	
2015	341 (8.4%)	20 (6.2%)	22 (8.4%)	
HCA dimensions				
Affordability score, mean (SD)	0.1 (0.9)	−0.7 (0.8)	−0.4 (1.0)	<0.001
Availability score, mean (SD)	0.0 (0.9)	−0.1 (0.9)	−0.0 (0.9)	0.121
Accessibility score, mean (SD)	−0.0 (0.5)	0.1 (0.4)	0.2 (0.4)	<0.001

NOTE: Cells with masked values such as <11, <15, <20, >20, and <50, have their cell values suppressed appropriately relative to the actual cell value, either because the cell has a value between 1 and 10, or to avoid backcalculation, in adherence with Centers for Medicare and Medicaid Services’ cell size suppression policy to protect patient privacy.

### EOL Care Quality Outcomes

**FIGURE 2:**
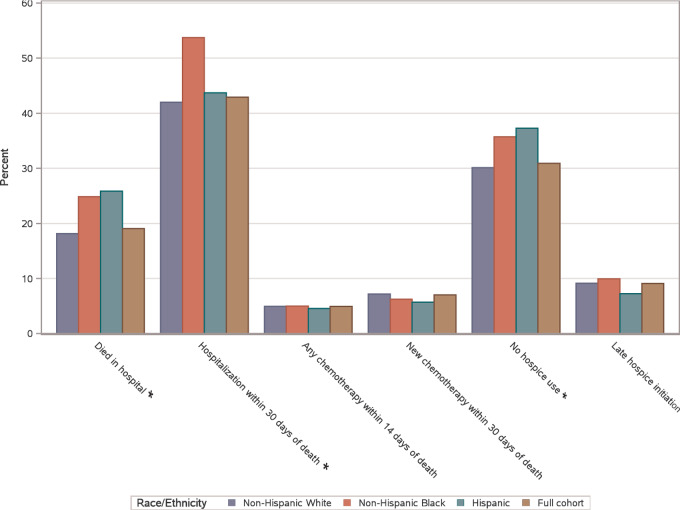
EOL care quality outcomes by patient race and ethnicity (*N* = 4,646). ICU stay and ER visit outcomes not shown due to cell suppression rules. Outcomes that differ by patient race/ethnicity (*χ*^2^ test *P* value <0.05) are denoted with an asterisk (*) on their label.

The distribution of the EOL care quality outcomes is shown in Fig. 2. Overall, 19.05% of the study cohort died in the hospital, with a relatively higher proportion of NHB (24.8%) and Hispanic (25.8%) patients, compared with NHW (18.1%; *P* <0.001). Similarly, 42.9% of patients were admitted to the hospital in the last month of life, with a higher proportion of NHB patients (53.7%) than NHW (42.0%) or Hispanic patients (43.7%). Approximately 7.0% of patients received systemic therapy in the last 14 days of life, and 4.9% initiated new chemotherapy in the last 30 days of life, with no differences by race or ethnicity. About one-third of the patients (30.9%) did not use hospice before death, with a composition of 30.1% NHW, 35.7% NHB, and 37.3% Hispanic patients, while late hospice use did not differ by race and ethnicity. ICU admissions in the last 30 days of life occurred in 14.9% of the patients in the cohort (Fig. 2; Supplementary Table S3). NHB and Hispanic patients had slightly higher average counts of poor-quality EOL care outcomes (NHB mean count = 1.59, Hispanic = 1.46, NHW = 1.29, *P* <0.001; Supplementary Table S1).

Minimally-adjusted models, in which we adjusted for patient characteristics and one HCA score, showed patterns that were consistent in magnitude and direction with the fully-adjusted models, with some deviations in statistical significance (see Supplementary Table S4). Therefore, results reported on below focus on the fully-adjusted multivariable models.

### HCA Dimension Scores and EOL Care Quality Outcomes

In the log-binomial regression models, HCA scores were associated with EOL care quality measures (Table 2). Higher affordability scores were associated with a 9% decreased risk of dying in the hospital [adjusted relative risk (aRR) 0.91, 95% confidence interval (CI): 0.84–0.98 per unit increase in score], and a 10% decreased risk of an ICU stay in the last 30 days of life (aRR 0.90, 95% CI: 0.83–0.98). Higher availability scores were associated with an 11% increased risk of dying in the hospital (aRR 1.11, 95% CI: 1.02–1.20), and a 7% increased risk of hospitalization in the last 30 days of life (aRR 1.07, 95% CI: 1.02–1.12). Higher accessibility scores were associated with a substantially increased risk of ICU stay (aRR 1.35, 95% CI: 1.14–1.60), a 12% reduced risk of not receiving hospice services prior to death (aRR 0.88, 95% CI: 0.80–0.95), and a 54% reduced risk of two or more ER visits in the last 30 days of life (OR 0.46, 95% CI: 0.35–0.62; Table 2). When examined as a count of poor-quality outcomes per patient, the HCA scores were not associated with higher outcome counts (Table 3).

**TABLE 2:** Estimates of the associations between HCA dimensions, patient race and ethnicity, and EOL care quality outcomes on entire study cohort (*N* = 4,646), subcohort of patients that died from ovarian cancer (*N* = 3,195), broader cohort of patients including those who had unknown stage/histology (*N* = 4,763), and same as overall cohort but with statistical adjustment for survival time (*N* = 4,646)

	*aRR (95% CI)*			
	Overall cohort (*N* = 4,646)	Died from ovarian cancer cohort (*N* = 3,195)	Including all stages and histologies along with unknown stage/ histology (*N* = 4,763)	Survival time adjustment (*N* = 4,646)
**Prevalent outcomes^a^**				
Died in the hospital				
Affordability score	**0.91 (0.84–0.98)**	0.92 (0.84–1.01)	0.9 (0.83–0.96)	0.94 (0.87–1.01)
Availability score	**1.11 (1.02–1.21)**	1.09 (0.98–1.20)	1.1 (1.01–1.19)	**1.11 (1.03–1.21)**
Accessibility score	0.91 (0.81–1.03)	0.85 (0.74–0.99)	0.94 (0.83–1.06)	0.92 (0.81–1.03)
Race (ref = NHW)				
NHB	**1.27 (1.03–1.57)**	1.33 (1.04–1.7)	1.26 (1.02–1.55)	1.22 (1.00–1.50)
Hispanic	1.23 (0.98–1.54)	1.35 (1.04–1.75)	1.20 (0.96–1.50)	1.17 (0.93–1.46)
Hospitalized in last 30 days of life				
Affordability score	0.99 (0.95–1.03)	1.02 (0.97–1.07)	0.99 (0.95–1.03)	1 (0.96–1.04)
Availability score	**1.07 (1.02–1.12)**	1.06 (1–1.12)	1.06 (1.02–1.11)	1.06 (1.02–1.11)
Accessibility score	1.04 (0.96–1.11)	1.02 (0.94–1.11)	1.05 (0.97–1.12)	1.04 (0.97–1.11)
Race (ref = NHW)				
NHB	**1.16 (1.03–1.30)**	1.24 (1.09–1.4)	1.17 (1.04–1.31)	**1.13 (1.01–1.26)**
Hispanic	1.01 (0.88–1.17)	1.06 (0.9–1.25)	1.02 (0.88–1.18)	0.98 (0.85–1.12)
Intensive care unit stay in last 30 days of life				
Affordability score	**0.90 (0.83–0.98)**	0.94 (0.85–1.04)	0.91 (0.84–0.98)	0.92 (0.85–1.00)
Availability score	1.04 (0.95–1.15)	0.98 (0.87–1.11)	1.04 (0.95–1.14)	1.04 (0.95–1.14)
Accessibility score	**1.35 (1.14–1.60)**	1.23 (1.01–1.49)	1.35 (1.15–1.6)	**1.35 (1.15–1.60)**
Race (ref = NHW)				
NHB	1.1 (0.86–1.41)	1.22 (0.92–1.62)	1.09 (0.86–1.4)	1.07 (0.84–1.37)
Hispanic	1.09 (0.83–1.42)	1.21 (0.89–1.64)	1.09 (0.84–1.41)	1.06 (0.81–1.38)
Did not initiate hospice prior to death				
Affordability score	0.97 (0.92–1.02)	0.93 (0.86–1.01)	0.96 (0.91–1.02)	0.96 (0.91–1.01)
Availability score	1.06 (1.00–1.12)	1.08 (0.99–1.18)	1.05 (1.00–1.12)	1.06 (1–1.12)
Accessibility score	**0.87 (0.80–0.95)**	**0.83 (0.73–0.93)**	**0.88 (0.81–0.96)**	**0.87 (0.8–0.95)**
Race (ref = NHW)				
NHB	**1.23 (1.04–1.44)**	**1.42 (1.15–1.76)**	**1.21 (1.03–1.41)**	**1.23 (1.05–1.44)**
Hispanic	1.11 (0.94–1.32)	**1.31 (1.04–1.64)**	1.1 (0.93–1.30)	1.12 (0.95–1.33)
**Less prevalent outcomes^a^**				
Initiated new chemotherapy agent within 30 days of death				
Affordability score	1.06 (0.92–1.22)	**1.32 (1.09–1.61)**	^b^	**1.24 (1.05–1.47)**
Availability score	1.06 (0.91–1.25)	0.83 (0.66–1.06)	^b^	0.94 (0.77–1.15)
Accessibility score	0.87 (0.7–1.1)	1 (0.7–1.44)	^b^	0.97 (0.72–1.31)
Race (ref = NHW)				
NHB	0.82 (0.5–1.34)	1.14 (0.62–2.12)	^b^	1.05 (0.60–1.83)
Hispanic	0.78 (0.45–1.36)	1.14 (0.57–2.27)	^b^	0.92 (0.49–1.72)
Received chemotherapy within 14 days of death				
Affordability score	1.06 (0.92–1.22)	1 (0.84–1.20)	^b^	1.06 (0.92–1.22)
Availability score	1.06 (0.91–1.25)	0.98 (0.80–1.20)	^b^	1.06 (0.91–1.25)
Accessibility score	0.87 (0.70–1.10)	0.78 (0.60–1.03)	^b^	0.87 (0.70–1.10)
Race (ref = NHW)				
NHB	0.82 (0.50–1.34)	0.88 (0.50–1.56)	^b^	0.82 (0.50–1.34)
Hispanic	0.78 (0.45–1.36)	0.86 (0.45–1.65)	^b^	0.78 (0.45–1.36)
Two or more ER visits in last 30 days of life				
Affordability score	0.87 (0.68–1.13)	0.83 (0.61–1.13)	0.88 (0.68–1.13)	0.87 (0.68–1.13)
Availability score	1.23 (0.96–1.58)	1.34 (0.99–1.8)	1.22 (0.95–1.57)	1.23 (0.96–1.58)
Accessibility score	**0.46 (0.35–0.62)**	**0.46 (0.32–0.66)**	**0.47 (0.35–0.62)**	**0.46 (0.35–0.62)**
Race (ref = NHW)				
NHB	1.58 (0.83–2.99)	2.19 (1.10–4.36)	1.6 (0.85–3.02)	1.58 (0.83–2.99)
Hispanic	0.7 (0.27–1.8)	0.59 (0.18–1.97)	0.71 (0.28–1.82)	0.7 (0.27–1.8)
Late initiation of hospice (within 3 days of death)				
Affordability score	1.13 (1–1.28)	1.1 (0.95–1.27)	1.13 (1–1.28)	1.13 (1.00–1.28)
Availability score	0.86 (0.74–1.01)	0.88 (0.74–1.05)	**0.85 (0.74–0.99)**	0.86 (0.74–1.01)
Accessibility score	1.02 (0.81–1.27)	1 (0.77–1.3)	1.04 (0.83–1.30)	1.02 (0.81–1.27)
Race (ref = NHW)				
NHB	1.13 (0.76–1.70)	1.37 (0.88–2.12)	1.13 (0.75–1.68)	1.13 (0.76–1.70)
Hispanic	0.73 (0.44–1.20)	0.66 (0.36–1.20)	0.73 (0.44–1.20)	0.73 (0.44–1.20)

Abbreviations: aRR, adjusted relative risk; HCA, health care access; NHB: non-Hispanic Black; NHW, non-Hispanic White.

^a^Fully-adjusted log-binomial regression model was performed on prevalent outcomes and adjusted relative risk (aRR, 95% CI) reported; while fully-adjusted logistic regression was performed on less prevalent outcomes and aOR (adjusted OR, 95% CI) reported. Models were additionally adjusted for age, tumor stage, tumor histology, patient comorbid conditions, geographic region of residence at diagnosis, and year of diagnosis.

^b^Chemotherapy outcomes were not assessed in sensitivity analyses with all ovarian cancer stages and histologies included along with patients with missing stage or histology as chemotherapy is not recommended for all stages and histologies.

**TABLE 3:** Adjusted count ratio estimates of the associations between HCA dimensions, patient race and ethnicity, and counts of EOL care quality outcomes as count ratios, from fully-adjusted^a^ negative binomial model

	Count ratio (95% CI)			
	Overall cohort (*N* = 4,646)	Died from ovarian cancer cohort (*N* = 3,195)	Including all stages and histologies along with unknown stage/ histology (*N* = 4,763)	Survival time adjustment (*N* = 4,646)
Affordability score (1 unit increase)	0.97 (0.93–1.01)	0.96 (0.92–1)	^b^	0.98 (0.94–1.02)
Availability score (1 unit increase)	1.05 (1.00–1.11)	1.05 (1.00–1.10)	^b^	1.05 (1.00–1.11)
Accessibility score (1 unit increase)	0.97 (0.90–1.04)	0.99 (0.92–1.06)	^b^	0.97 (0.90–1.04)
Race (ref = NHW)				
NHB	**1.19 (1.04–1.36)**	**1.20 (1.05–1.36)**	^b^	**1.17 (1.02–1.34)**
Hispanic	1.05 (0.90–1.22)	1.06 (0.92–1.22)	^b^	1.03 (0.89–1.2)

Abbreviations: HCA: health care access; NHW: non-Hispanic White; NHB: non-Hispanic Black.

^a^Negative binomial model adjusted for age, tumor stage, tumor histology, patient comorbid conditions, geographic region of residence at diagnosis, and year of diagnosis.

^b^Count outcomes were not assessed in sensitivity analyses with all ovarian cancer stages and histologies included along with patients with missing stage or histology as chemotherapy is not recommended for all stages and histologies. As chemotherapy outcomes could not be assessed, the count outcomes would not be directly comparable to the main analyses.

### Racial Disparities and EOL Care Quality Outcomes

After full model adjustment, NHB patients remained at an increased risk of common poor EOL care quality outcomes compared with NHW patients, including in-hospital death (aRR 1.26, 95% CI: 1.03–1.57), hospitalization in the last 30 days of life (aRR 1.16, 95% CI: 1.03–1.30), and no hospice care prior to death (aRR 1.23, 95% CI: 1.04–1.44; Table 2). There were no differences in the odds of less common EOL care outcomes (Table 2). However, NHB patients still had 19% higher counts of poor-quality EOL care outcomes when compared with NHW patients after adjustment for HCA scores and demographic and clinical characteristics (count ratio:1.19, 95% CI: 1.04–1.36; Table 3). Moreover, in the fully-adjusted models, Hispanic patients did not differ from NHW patients in all EOL care quality outcomes (Table 2).

### Interactions Between HCA Dimension Scores and Race and Ethnicity

There was no evidence of a statistical interaction between affordability or availability scores and patient race and ethnicity for any outcome. However, the association between accessibility score and receipt of chemotherapy within 14 days of death differed with race and ethnicity. Higher accessibility scores were associated with a decreased risk of chemotherapy within 14 days of death among Hispanic patients compared with NHW patients, and a nonsignificant increased risk among NHB patients (Supplementary Table S5). There was no evidence of an interaction among accessibility, patient race, and ethnicity for any other outcome.

### Sensitivity Analyses

All three sensitivity analyses produced results that were consistent overall in direction with results from the primary analyses, with some deviation in magnitude and statistical significance (Table 2). Magnitude of racial disparities seemed to increase when survival time was adjusted, as well as for the study cohort that was reduced to patients that died from ovarian cancer. For example, in died with ovarian cancer and survival time adjustment analyses, and with adjustment for HCA dimensions, Hispanics showed a 35% and 17% higher risk of death in the hospital relative to NHW patients, respectively.

## Discussion

In this retrospective study of NHB, NHW, and Hispanic patients with ovarian cancer in the SEER-Medicare database (2008–2015), each HCA dimension examined was significantly associated with different aspects of EOL care. Affordability was associated with a decreased risk of ICU stay and hospitalization in the last month of life. Availability was associated with an increased risk of hospitalization in the last month of life and in-hospital mortality. Accessibility was associated with an increased risk of ICU admission, a decreased risk of no hospice use, and a decreased risk of two or more ER visits in the last month of life. Moreover, while HCA dimensions explained differences in EOL quality between Hispanic and NHW patients in the primary analysis, racial differences persisted for NHB patients, who remained at a 15% to 26% increased risk of poor EOL care quality after accounting for health care affordability, accessibility, and availability. Among patients who died from ovarian cancer or when survival time was adjusted for, racial disparities persisted for NHB and Hispanic patients even after adjustment for HCA dimensions. These results highlight persistent disparities in the quality of EOL care for patients with ovarian cancer and the unique contribution of HCA dimensions, while justifying the need to examine other, yet unexplored HCA dimensions. Such dimensions include accommodation and acceptability, which address patients’ perceptions about convenience of care and satisfaction with provider interactions and health care received (21).

Previous studies have described racial disparities in EOL care among patients with ovarian cancer (9, 12, 13). Fairfield and colleagues (2012) reported that non-White patients had a 44% increased odds of lack of hospice use in a SEER-Medicare cohort of patients with ovarian cancer diagnosed between 2001 and 2005 (13). Similarly, Taylor and colleagues (2017) showed that among older patients with ovarian cancer in Texas who died between 2000 and 2012, Black patients had 36% lower odds of enrolling and dying in hospice care, 120% higher odds of having more than one ER visit, and 113% higher odds of having a life-extending procedure (9). Most recently, Mullins and colleagues (2021) found that Black patients with ovarian cancer diagnosed between 2007 and 2016, similar to our study, had double the odds of White women for >1 ER visit in the last month of life compared with White women (12). In this study, we showed similar higher risks for poor EOL quality care among NH Black and Hispanic patients. Our results are consistent with prior studies and further advance the body of knowledge on this subject by demonstrating that racial disparities, albeit attenuated, persist when major drivers of social inequities related to access, affordability, and availability are accounted for.

We observed that higher affordability, measured at the neighborhood level with indicators of poverty rates and educational attainment at the time of ovarian cancer diagnosis, was associated with better quality EOL care outcomes. Patients in low-income neighborhoods experience higher levels of socioeconomic deprivation, which may directly contribute to the observed affordability pattern, through higher financial distress, higher physical symptom burden, poorer health and function, failure of existing health care resources and services to meet low-income patients’ complex needs leading to repeated utilizations, and an inherent preference for more intensive EOL care (30). Conversely, patients with ovarian cancer in affluent neighborhoods may benefit from access to higher health care quality, including better pain and symptom management (31), corresponding to less comorbidity burden and outcome severity, thus reducing the need for health care utilization during EOL. Health literacy differences in socioeconomic status may also play a role, (32) with patients in high health care affordability neighborhoods more likely to have higher health literacy levels and be involved in health care decision-making through advance care planning directives that limit intensive interventions during EOL (33, 34). This finding implies that a fundamental step in minimizing disparities in EOL care involves the real-world adoption of a range of effective policies including effective pain management and symptom management prior to EOL, services that can be supported with equitable HCA (35).

Moreover, we observed that barriers to EOL care for NHB patients with ovarian cancer likely transcended the physical availability of specialist providers and the geographic proximity of health care facilities. On average, NHB and Hispanic patients had higher accessibility and availability scores than NHW patients with ovarian cancer, which was associated with poor-quality EOL care outcomes. Consequently, our results suggest more acute disparities in EOL care for NHB patients not necessarily mitigated by HCA, as further buttressed by opposing associations for accessibility, with a decreased risk of chemotherapy in the last 2 weeks of life for Hispanics but a nonsignificant increased risk for NHB. We cannot discount the possibility that patients with NHB may be sicker on average, with a higher comorbidity burden, as observed in our study. Coupled with complex ovarian cancer disease and symptom management needs, NHB patients may require ER or inpatient hospitalizations for treatment (16, 36, 37). Moreover, due to the inability to afford out-of-pocket or informal costs not covered by Medicare, including travel and time of nonpaid caregivers, high-quality EOL care such as hospice use may be beyond the reach of many NHB patients (30, 38, 39). NHB patients may actively pursue Medicare-covered rehabilitation services to avoid out-of-pocket hospice costs (40). Therefore, our findings suggest that aggressive EOL care for NHB patients may potentially be mitigated by addressing gaps in pain and symptom management, improving health care affordability, health literacy, and access to resources for advance care planning and hospice care prior to EOL.

Additional factors more likely captured by the yet unexplored HCA dimensions of accommodation and acceptability may also contribute to low-quality EOL care for NHB patients with ovarian cancer. Given the history of exploitation and unethical medical experimentation that Black Americans have experienced (1), the persistent pattern of NHB patients embracing aggressive EOL care may reflect mistrust of the health system. Moreover, there might be an actionable role for social networks and caregivers in improving receipt of high-quality EOL care among NHB patients with ovarian cancer, as evidence suggests that intensity and size of social capital can influence EOL decisions and access to EOL resources (41). The lack of culturally sensitive palliative care (1) and patient preferences that are at odds with quality EOL care (1), physician bias (1, 3), and health literacy levels of NHB patients (1, 42), are all critical challenges that highlight the need for system-wide interventions for patients, families, care teams, and support groups (43), with the goal of better quality EOL care (1).

There are certain limitations in interpreting our findings. First, the study cohort was comprised of NHW, NHB, and Hispanic ovarian cancer FFS Medicare beneficiaries ages ≥65 years, which restricts the extent to which results can be generalized to younger patients or those enrolled in managed care (Medicare part C), or those of other races. Second, the three HCA dimensions considered were measured at the area level, leaving individual-level associations with EOL care outcomes unexplored. Moreover, our HCA scores were measured in generic terms, not capturing EOL-relevant information such as hospice bed supply and number of palliative care physicians. Third, our study cohort was heterogeneous in histologic subtypes, and given known variation of ovarian cancer survival with subtype (44), future larger studies with more representative distribution of histologic subtypes will be better placed to investigate how disparities in EOL care may vary with subtypes. Finally, reported patterns of racial disparities in EOL care among patients with ovarian cancer may be reflective of the study period (2008–2015); however, the results are unlikely to be markedly different from more recent data because the structural barriers and challenges that underpin racial disparities are yet to be resolved on a large scale. Nevertheless, this study has the advantage of using a large, population-based sample with empirical measures of EOL care estimated from claims data and extensive measures of HCA dimensions, and makes the novel contribution of characterizing key drivers of EOL care disparities among patients with ovarian cancer.

In conclusion, in a sample of older decedent patients with ovarian cancer, racial differences in EOL care were attenuated, but persisted after adjusting for HCA dimensions. Further investigations on accommodation and acceptability HCA dimensions can shed light on poorly explored barriers to high-quality EOL. Our findings demonstrate the need for strategies to standardize the receipt of supportive care, palliative care, and EOL care for terminally ill patients with ovarian cancer irrespective of race and ethnicity.

## Supplementary Material

Supplementary Methods Additional description of data sources and statistical methods

Supplementary Table 1 Patient, census tract, and regional level variables measuring dimensions of healthcare affordability, availability and accessibility

Supplementary Table 2 Factor Loadings From the 2-Stage 3-Factor CFA Solution

Supplementary Table 3 EOL Care Quality Outcomes by Patient Race/Ethnicity

Supplementary Table 4 Relative risk ratio estimates of the associations between HCA dimensions, patient race/ethnicity, and EOL care quality outcomes

Supplementary Table 5 Relative risk ratio (RR) for receipt of any chemotherapy within 14 days of death with interaction term between accessibility score and patient race

## References

[1] Bazargan M , Bazargan-Hejazi S. Disparities in palliative and hospice care and completion of advance care planning and directives among non-Hispanic Blacks: a scoping review of recent literature. Am J Hosp Palliat Care 2021;38:688–718. 33287561 10.1177/1049909120966585 PMC8083078

[2] Meier EA , Gallegos JV, Thomas LP, Depp CA, Irwin SA, Jeste DV. Defining a good death (Successful Dying): literature review and a call for research and public dialogue. Am J Geriatr Psychiatry 2016;24:261–71. 26976293 10.1016/j.jagp.2016.01.135 PMC4828197

[3] Nelson KE , Wright R, Peeler A, Brockie T, Davidson PM. Sociodemographic disparities in access to hospice and palliative care: an integrative review. Am J Hosp Palliat Care 2021;38:1378–90. 33423532 10.1177/1049909120985419 PMC8514114

[4] Smith CB , Phillips T, Smith TJ. Using the new ASCO clinical practice guideline for palliative care concurrent with oncology care using the TEAM approach. Am Soc Clin Oncol Educ Book 2017;37:714–23. 28561696 10.1200/EDBK_175474

[5] Lewin SN , Buttin BM, Powell MA, Gibb RK, Rader JS, Mutch DG, . Resource utilization for ovarian cancer patients at the end of life: how much is too much? Gynecol Oncol 2005;99:261–6. 16140364 10.1016/j.ygyno.2005.07.102

[6] Cheung MC , Earle CC, Rangrej J, Ho TH, Liu N, Barbera L, . Impact of aggressive management and palliative care on cancer costs in the final month of life. Cancer 2015;121:3307–15. 26031241 10.1002/cncr.29485 PMC4560956

[7] MedPAC. Report to the congress: Medicare payment policy. Washington, DC; 2020.

[8] Emanuel EJ . The status of end-of-life care in the United States: the glass is half full. JAMA 2018;320:239–41. 30027232 10.1001/jama.2018.10062

[9] Taylor JS , Rajan SS, Zhang N, Meyer LA, Ramondetta LM, Bodurka DC, . End-of-life racial and ethnic disparities among patients with ovarian cancer. J Clin Oncol 2017;35:1829–35. 28388292 10.1200/JCO.2016.70.2894 PMC5455594

[10] Perry LM , Walsh LE, Horswell R, Miele L, Chu S, Melancon B, . Racial disparities in end-of-life care between Black and White adults with metastatic cancer. J Pain Symptom Manage 2021;61:342–9. 32947018 10.1016/j.jpainsymman.2020.09.017 PMC8100959

[11] Ornstein KA , Roth DL, Huang J, Levitan EB, Rhodes JD, Fabius CD, . Evaluation of racial disparities in hospice use and end-of-life treatment intensity in the REGARDS cohort. JAMA Netw Open 2020;3:e2014639. 32833020 10.1001/jamanetworkopen.2020.14639 PMC7445597

[12] Mullins MA , Ruterbusch JJ, Clarke P, Uppal S, Wallner LP, Cote ML. Trends and racial disparities in aggressive end-of-life care for a national sample of women with ovarian cancer. Cancer 2021;127:2229–37. 33631053 10.1002/cncr.33488 PMC8195844

[13] Fairfield KM , Murray KM, Wierman HR, Han PKJ, Hallen S, Miesfeldt S, . Disparities in hospice care among older women dying with ovarian cancer. Gynecol Oncol 2012;125:14–8. 22138230 10.1016/j.ygyno.2011.11.041

[14] Algu K . Denied the right to comfort: racial inequities in palliative care provision. EClinicalMedicine 2021;34:100833. 33937727 10.1016/j.eclinm.2021.100833 PMC8079452

[15] Wright AA , Hatfield LA, Earle CC, Keating NL. End-of-life care for older patients with ovarian cancer is intensive despite high rates of hospice use. J Clin Oncol 2014;32:3534–9. 25287831 10.1200/JCO.2014.55.5383 PMC4209104

[16] Peres LC , Schildkraut JM. Racial/ethnic disparities in ovarian cancer research. Adv Cancer Res 2020;146:1–21. 32241384 10.1016/bs.acr.2020.01.002

[17] Brown AJ , Sun CC, Prescott LS, Taylor JS, Ramondetta LM, Bodurka DC. Missed opportunities: patterns of medical care and hospice utilization among ovarian cancer patients. Gynecol Oncol 2014;135:244–8. 25192878 10.1016/j.ygyno.2014.08.039 PMC4372337

[18] Elk R , Felder TM, Cayir E, Samuel CA. Social inequalities in palliative care for cancer patients in the United States: a structured review. Semin Oncol Nurs 2018;34:303–15. 30146346 10.1016/j.soncn.2018.06.011 PMC7233309

[19] Akinyemiju T , Deveaux A, Wilson L, Gupta A, Joshi A, Bevel M, . Ovarian cancer epidemiology, healthcare access and disparities (ORCHiD): methodology for a population-based study of Black, Hispanic and White patients with ovarian cancer. BMJ Open 2021;11:e052808. 10.1136/bmjopen-2021-052808 PMC8491419 34607872

[20] Penchansky R , Thomas JW. The concept of access: definition and relationship to consumer satisfaction. Med Care 1981;19:127–40. 7206846 10.1097/00005650-198102000-00001

[21] Thomas JW , Penchansky R. Relating satisfaction with access to utilization of services. Med Care 1984;22:553–68. 6738145 10.1097/00005650-198406000-00006

[22] Warren JL , Klabunde CN, Schrag D, Bach PB, Riley GF. Overview of the SEER-Medicare data: content, research applications, and generalizability to the United States elderly population. Med Care 2002;40:IV-3-18. 10.1097/01.MLR.0000020942.47004.03 12187163

[23] Koshiyama M , Matsumura N, Konishi I. Recent concepts of ovarian carcinogenesis: type I and type II. Biomed Res Int 2014;2014:934261. 24868556 10.1155/2014/934261 PMC4017729

[24] Morden NE , Chang C-H, Jacobson JO, Berke EM, Bynum JPW, Murray KM, . End-of-life care for Medicare beneficiaries with cancer is highly intensive overall and varies widely. Health Aff 2012;31:786–96. 10.1377/hlthaff.2011.0650 PMC3338099 22492896

[25] Committee on Approaching Death: Addressing Key End of Life Issues, Institute of Medicine. Dying in America: improving quality and honoring individual preferences near the end of life. Washington (DC): National Academies Press (US); 2015. 25927121

[26] Matz M , Coleman MP, Sant M, Chirlaque MD, Visser O, Gore M, . The histology of ovarian cancer: worldwide distribution and implications for international survival comparisons (CONCORD-2). Gynecol Oncol 2017;144:405–13. 27931752 10.1016/j.ygyno.2016.10.019 PMC6195192

[27] Klabunde CN , Warren JL, Legler JM. Assessing comorbidity using claims data: an overview. Med Care 2002;40:IV-26-35. 10.1097/00005650-200208001-00004 12187165

[28] Quan H , Sundararajan V, Halfon P, Fong A, Burnand B Luthi J-C, . Coding algorithms for defining comorbidities in ICD-9-CM and ICD-10 administrative data. Med Care 2005;43:1130–9. 16224307 10.1097/01.mlr.0000182534.19832.83

[29] Gupta A , Chen Q, Wilson LE, Huang B, Pisu M, Liang M, . Factor analysis of health care access with ovarian cancer surgery and gynecologic oncologist consultation. JAMA Netw Open 2023;6:e2254595. 36723938 10.1001/jamanetworkopen.2022.54595 PMC9892953

[30] Bowers SP , Chin M, O'Riordan M, Carduff E. The end of life experiences of people living with socio-economic deprivation in the developed world: an integrative review. BMC Palliative Care 2022;21:193. 36335335 10.1186/s12904-022-01080-6 PMC9636719

[31] Ward E , Jemal A, Cokkinides V, Singh GK, Cardinez C, Ghafoor A, . Cancer disparities by race/ethnicity and socioeconomic status. CA Cancer J Clin 2004;54:78–93. 15061598 10.3322/canjclin.54.2.78

[32] Stormacq C , Van den Broucke S, Wosinski J. Does health literacy mediate the relationship between socioeconomic status and health disparities? Integrative review. Health Promot Int 2019;34:e1–17. 30107564 10.1093/heapro/day062

[33] Zhang B , Wright AA, Huskamp HA, Nilsson ME, Maciejewski ML, Earle CC, . Health care costs in the last week of life: associations with end-of-life conversations. Arch Intern Med 2009;169:480–8. 19273778 10.1001/archinternmed.2008.587 PMC2862687

[34] Volandes AE , Paasche-Orlow M, Gillick MR, Cook EF, Shaykevich S, Abbo ED, . Health literacy not race predicts end-of-life care preferences. J Palliat Med 2008;11:754–62. 18588408 10.1089/jpm.2007.0224

[35] Wachterman MW , Sommers BD. Dying poor in the US—disparities in end-of-life care. JAMA 2021;325:423–4. 33528526 10.1001/jama.2020.26162

[36] Anyanwu MC , Ohamadike O, Wilson LE, Meernik C, Huang B, Pisu M, . Race, affordability and utilization of supportive care in ovarian cancer patients. J Pain Symptom Manage 2022;64:537–45. 36058401 10.1016/j.jpainsymman.2022.08.021 PMC10083071

[37] Broekman KE , van der Aa MA, Nijman HW, Jalving M, Reyners AKL. End-of-life care for patients with advanced ovarian cancer in the Netherlands: a retrospective registry-based analysis. Gynecol Oncol 2022;166:148–53. 35644730 10.1016/j.ygyno.2022.04.017

[38] Taylor DH . The effect of hospice on medicare and informal care costs: the U.S. experience. J Pain Symptom Manage 2009;38:110–4. 19615635 10.1016/j.jpainsymman.2009.04.003

[39] Drutchas A , Baughman AW, Ritchie CS. The hospice paradox: how Medicare fails Americans at the end of life. Health Affairs Forefront; 2022.

[40] Fausto J . Filling the void: making end-of-life care a medicare “skilled need” to span the spectrum of care needed for high-quality, end-of-life care in the United States; 2018.

[41] Kumar V , Ankuda CK, Aldridge MD, Husain M, Ornstein KA. Family caregiving at the end of life and hospice use: a national study of Medicare beneficiaries. J Am Geriatr Soc 2020;68:2288–96. 32602571 10.1111/jgs.16648 PMC7718293

[42] Wang SY , Hsu SH, Huang S, Doan KC, Gross CP, Ma X. Regional practice patterns and racial/ethnic differences in intensity of end-of-life care. Health Serv Res 2018;53:4291–309. 29951996 10.1111/1475-6773.12998 PMC6232508

[43] Payne R . Racially associated disparities in hospice and palliative care access: acknowledging the facts while addressing the opportunities to improve. J Palliat Med 2016;19:131–3. 26840847 10.1089/jpm.2015.0475

[44] Zhou J , Wu S-G, Wang J, Sun J-Y, He Z-Y, Jin X, . The effect of histological subtypes on outcomes of stage IV epithelial ovarian cancer. Front Oncol 2018;8:577. 30564556 10.3389/fonc.2018.00577 PMC6288295

