# Supplementary material for: Health Care Access Dimensions and Racial Disparities in End-of-Life Care Quality among Patients with Ovarian Cancer
Source: Cancer Res Commun. 2024 Mar 18;4(3):811–21. doi: 10.1158/2767-9764.CRC-23-0283 (PMC10946308; doi:10.1158/2767-9764.CRC-23-0283)
Supplement: Supplementary Methods — Additional description of data sources and statistical methods [file crc-23-0283-s01.docx]

**Supplementary Methods**

*Assignment of primary provider and hospital treatment facility:* A patient’s primary cancer treatment provider was identified as the provider listed on the highest number of the patient’s outpatient, carrier, home health, and hospice claims listing a cancer diagnosis. Physician specialties were determined from Medicare claims files using Health Care Financing Administration (HCFA) specialty codes. Ties between physicians were broken by prioritizing physician specialties of interest (gynecologic oncology, medical oncology, hematology/oncology, or surgical oncology) and claim date closest to the ovarian cancer (OC) diagnosis date. The patient’s primary treating hospital in the year the patient was diagnosed was defined as the facility at which the patient had the majority of inpatient and outpatient claims in that calendar year. In the case of ties, priority was given to facilities with records in the SEER-Medicare Hospital File.

*Measures of healthcare affordability:* Measures of healthcare affordability included dual enrollment in Medicaid, census tract-level measures of socioeconomic status (SES), and county-level health insurance coverage. A patient’s dual Medicaid enrollment status in the 12 months prior to OC diagnosis was sourced from the SEER-Medicare dataset, as were the following SES indicators of the patient’s residential census tract at the time of diagnosis drawn from data from the US Census Bureau’s American Community Survey: median per capita income, percentage of Black residents, percentage of adults 25+ with less than a high school education, percentage of households with incomes below the poverty level, and percentage of adults 25+ with a college degree. Census tract SES characteristics were categorized into quartiles, and included as a binary variable in models (highest quartile versus lower three quartiles). Federal Information Processing Standards (FIPS) codes for the patient’s county and state of residence and the patient’s year of diagnosis were used to link to the US Census Bureau’s Small Area Health Insurance Estimates 2008-2018 American Community Survey-Based Estimates datasets (https://www.census.gov/data/datasets/time-series/demo/sahie/estimates-acs.html) to obtain the estimated percentage of county residents without health insurance in the year of the patient’s diagnosis.

*Measures of healthcare availability:* Healthcare availability metrics for the patient’s county and healthcare referral regions were linked to SEER-Medicare data using year of diagnosis, county and state FIPS codes, and patient zip codes from the Area Healthcare Resources File and the Dartmouth Atlas Project. County-level metrics were drawn from the publicly available Area Healthcare Resource Files provided by the Health Resources and Services Administration (<https://data.hrsa.gov/data/download>). County-level linked measures were calculated as number per 1,000 population and included number of hospitals, number of primary care providers, and number of obstetricians-gynecologists (Ob-Gyns). Hospital referral region (HRR)-level availability metrics derived from Medicare and Medicaid data from the Dartmouth Atlas Project (<https://atlasdata.dartmouth.edu/downloads>) were linked using patient zip code and year of diagnosis. HRR data captures the characteristics of the regional markets for tertiary healthcare systems. HRR-level availability metrics of interest for the patient’s year of diagnosis were acute care beds available per 1K population, physicians per 100K population, primary care physicians per 100K population, Hematologists/Oncologists per 100K population, Ob-Gyns per 100K women aged 15-44, percentage of Medicare beneficiaries that died, percentage of beneficiaries seeing a primary care physician (PCP) that year, discharges for ambulatory sensitive conditions per 1K population, hospital discharge 30 day readmission rates, and hospital discharge 30 day return to emergency room (ER) rates. For metrics without data available for each calendar year, the information was imputed from the most proximate year available to the patient’s diagnosis within five years. The National Cancer Institute (NCI) hospital file was used to determine facility-associated availability metrics including the hospital’s ownership, affiliation with a medical school, NCI Cancer Center designation critical access status, and number of beds in the year of the patient’s cancer diagnosis. If the hospital’s information was missing in a calendar year, the information was imputed as the highest availability value for the hospital recorded in the study time period.

*Measures of healthcare accessibility*: six variables, including patient residence at diagnosis in a metropolitan area, straight line geographic distance in miles from a patient’s residence to their main treatment hospital, patient's main treatment hospital’s location in an urban or rural area, were abstracted to represent healthcare Accessibility (see Supplementary Table 1). SEER provides urban or rural status based on Rural-Urban Commuting Area (RUCA) codes, and distance was calculated as straight line distance from the center of a patient's zip code to the center of treatment facility zip code.

*Factor analysis and creation of HCA factor scores*

We used the Penchansky and Thomas framework of healthcare access to guide our selection of variables representing the hypothesized latent constructs of healthcare access (affordability, availability, and accessibility) to include in our analysis using a two-stage confirmatory factor analysis approach. First, factor analysis was conducted for each *a priori* grouping of HCA dimension measures (Affordability, Availability, and Accessibility), then variables with significant loadings in the three preliminary models were carried forward into one final combined model. Two variables measuring number of specialists available (gynecologic oncologists and OB-GYNS) had high correlation efficiency, thus the gynecologic oncologist variable was excluded. A total of 18 HCA dimension measures were carried over and loaded into the second stage factor analysis. There was a clear separation for each of the three hypothesized factors (representing Affordability, Availability, and Accessibility) on the factor analysis scree plot, and each factor captured the majority of measures for a hypothesized HCA domain. We next conducted reliability tests and assessed model fit for these selected factors. Based on the reliability tests, we adjusted our final factor model by excluding the number of hospitals per 1K county population variable, which resulted in improved reliability metrics for the accessibility domain. The final factor model comprised a total of 13 variables loading onto the 3 factors, with close to 89% of the sample variance was explained. We also conducted exploratory factor analysis to agnostically determine factor structure for HCA domains. However, the 3-factor model did not demonstrate a simple and clear structure with respect to which variables loaded together on each factor, and factors 2 and 3 had low reliability scores when assessed using Cronbach’s alpha coefficient. Therefore, to improve interpretability and reliability of the factor scores, we relied on the confirmatory factor analysis approach.

Estimated factor scores for each HCA domain were created using PROC FACTOR to generate a linear composite of optimally weighted variables under analysis. To test heterogeneity of the associations by patient race and ethnicity, values of factor scores were stratified by race and ethnicity. Factor weighted sum scores were compared for each factor across patient race and ethnicity. Scores were centered at zero, with values ranging from approximately -3 to 4, with negative values representing the lowest scores for the dimension (i.e. low affordability), and positive scores representing higher scores for the dimension. Factor analyses were conducted using SAS version 9.4 (2013, SAS Institute, Cary, NC).
