# Supplementary material for: Health Care Access Dimensions and Racial Disparities in End-of-Life Care Quality among Patients with Ovarian Cancer
Source: Cancer Res Commun. 2024 Mar 18;4(3):811–21. doi: 10.1158/2767-9764.CRC-23-0283 (PMC10946308; doi:10.1158/2767-9764.CRC-23-0283)
Supplement: Supplementary Table 1 — Patient, census tract, and regional level variables measuring dimensions of healthcare affordability, availability and accessibility [file crc-23-0283-s02.docx]

| **Supplementary Table 1:** 35 patient, census tract, and regional level variables measuring dimensions of healthcare affordability, availability and accessibility put into confirmatory factor analysis |
| --- |
| **AFFORDABILITY MEASURES** |
| *Categorical Variables* |
| Patient is dual enrolled in Medicaid and Medicare |
| Patient's primary hospital eligibility for disproportionate share payments |
| *Continuous Variables* |
| Census tract at diagnosis: Percent Black residents |
| Census tract at diagnosis: Percent persons 25+ with at least 4 years of college |
| Census tract at diagnosis: Median household income |
| Census tract at diagnosis: Percent persons 25+ with <high school education |
| Census tract at diagnosis: Per capita income for Census tract |
| Census tract at diagnosis: Percent persons 25+ with some college education |
| Census tract at diagnosis: Percent of households below poverty line |
| County level: Percent of residents without health insurance |
|  |
| **ACCESSIBILITY MEASURES** |
| *Categorical Variables* |
| Patient lives in metropolitan area |
| Patient lives in a metropolitan or metropolitan-adjacent area |
| Patient lives in rural area |
| Patient's main hospital is rural primary hospital |
| *Continuous Variables* |
| Straight line geographic distance from patient residential zip code to patient's main hospital zip code |
| County level: # hospitals per 1K residents in patient’s county in year of diagnosis |
|  |
| **AVAILABILITY MEASURES** |
| *Categorical Variables* |
| Patient's main hospital teaching status |
| Patient's main hospital NCI cancer center designation |
| Clinical |
| Comprehensive |
| Patient's main hospital is member of NCI gynecologic oncology group |
| Specialty of patient’s primary cancer physician |
| General surgery |
| Gynecologic oncology |
| Hematology/oncology/medical oncology |
| Internal medicine |
| No primary |
| Ob-Gyn |
| Other |
| Pathology/other oncology |
| Primary/general |
| Surgical oncology |
| *Continuous Variables* |
| Patient's main hospital number of beds |
| HRR level: Discharges for ambulatory sensitive conditions per 1K population |
| HRR level: Hematologists/oncologists per 100K residents |
| HRR level: Percentage of Medicare beneficiaries that died in year of diagnosis |
| HRR level: Hospital-based physicians per 100K residents (2011) |
| HRR level: Ob-Gyn per 100K women |
| HRR level: Percentage of Medicare beneficiaries seeing a PCP that year |
| HRR level: PCPs per residents |
| HRR level: Hospital discharge 30-day return to ER rates (%) |
| HRR-level: 30 days hospital readmission rates (%) |
| HRR level: Physicians per 100K residents |
| HRR level: Surgeons per 100K residents |
| County level: # Gynecologic oncologists per 1K residents in year of diagnosis |
| County level: # Ob-Gyns seeing patients per 1K residents |
| County level: # PCPs per 1K residents |
| Abbreviations: standard deviation (SD); obstetrician-gynecologist (Ob-Gyn); primary care physician (PCP); National Cancer Institute (NCI); emergency room (ER); ovarian cancer (OC); hospital referral region (HRR). |
