# Supplementary material for: Health Care Access Dimensions and Racial Disparities in End-of-Life Care Quality among Patients with Ovarian Cancer
Source: Cancer Res Commun. 2024 Mar 18;4(3):811–21. doi: 10.1158/2767-9764.CRC-23-0283 (PMC10946308; doi:10.1158/2767-9764.CRC-23-0283)
Supplement: Supplementary Table 2 — Factor Loadings From the 2-Stage 3-Factor CFA Solution [file crc-23-0283-s03.docx]

| **Supplementary Table 2**: Factor Loadings From the 2-Stage 3-Factor CFA Solution; table sourced from Gupta, Chen, Wilson, Huang, Pisu, Liang, Previs, Moss, Ward, Schymura, Berchuck and Akinyemiju ^1^ | |
| --- | --- |
| **Factor**[**^a^**](https://www.ncbi.nlm.nih.gov/pmc/articles/PMC9892953/table/zoi221546t3/?report=objectonly#zoi221546t3n1) | **Standardized factor loadings** |
| **Availability: HRR-level variables** |  |
| Hematologists or oncologists per 100 000 residents[^b^](https://www.ncbi.nlm.nih.gov/pmc/articles/PMC9892953/table/zoi221546t3/?report=objectonly#zoi221546t3n2) | 0.76 |
| Hospital-based physicians per 100 000 residents[^b^](https://www.ncbi.nlm.nih.gov/pmc/articles/PMC9892953/table/zoi221546t3/?report=objectonly#zoi221546t3n2) | 0.63 |
| HRR: Primary care physicians per 100 000 residents[^b^](https://www.ncbi.nlm.nih.gov/pmc/articles/PMC9892953/table/zoi221546t3/?report=objectonly#zoi221546t3n2) | 0.88 |
| HRR: Total physicians per 100 000 residents[^b^](https://www.ncbi.nlm.nih.gov/pmc/articles/PMC9892953/table/zoi221546t3/?report=objectonly#zoi221546t3n2) | 1.00 |
| HRR: Surgeons per 100 000 residents[^b^](https://www.ncbi.nlm.nih.gov/pmc/articles/PMC9892953/table/zoi221546t3/?report=objectonly#zoi221546t3n2) | 0.75 |
| **Affordability: census tract–level variables at diagnosis** |  |
| Census tract at diagnosis: % residents 25+ with at least 4 y of college | 0.88 |
| Census tract at diagnosis: median household income | 0.85 |
| Census tract at diagnosis: % residents 25+ with <12 y education | −0.83 |
| Census tract at diagnosis: mean per capita income | 0.89 |
| Census tract at diagnosis: % households below poverty line | −0.74 |
| **Accessibility variables** |  |
| Patient residence in a metropolitan or metropolitan-adjacent area | 0.63 |
| Patient lives in metropolitan area | 0.78 |
| Patient’s main hospital is designated rural primary hospital | −0.52 |
| County-level hospitals, No. per 1000 residents in year of diagnosis | −0.67 |
| ^a^ Model fit: comparative fit index: 0.90; Tucker-Lewis Index: 0.88; standardized root-mean-square residual: 0.07.  ^b^ Data from 2011.  Abbreviations: CFA, confirmatory factor analysis; HRR, hospital referral region. | |
