# Supplementary material for: Health Care Access Dimensions and Racial Disparities in End-of-Life Care Quality among Patients with Ovarian Cancer
Source: Cancer Res Commun. 2024 Mar 18;4(3):811–21. doi: 10.1158/2767-9764.CRC-23-0283 (PMC10946308; doi:10.1158/2767-9764.CRC-23-0283)
Supplement: Supplementary Table 3 — EOL Care Quality Outcomes by Patient Race/Ethnicity [file crc-23-0283-s04.docx]

| **Supplementary Table 3:** EOL Care Quality Outcomes by Patient Race/Ethnicity (N=4,646) | | | | |
| --- | --- | --- | --- | --- |
| **Variable** | **NHW** | **NHB** | **Hispanic** | **p-value** |
| N | 4,061 | 322 | 263 |  |
| Count: poor EOL quality end-of-life measures |  |  |  |  |
| Median poor quality measure count (25^th^-75^th^ percentile) | 1 (0-2) | 1 (0-3) | 1 (0-3) | **0.009** |
| Mean poor quality measure count (SE) | 1.29 (0.02) | 1.59 (0.08) | 1.46 (0.09) | **<0.001** |
| Poor EOL Care Quality Measures |  |  |  |  |
| Any new chemotherapy agent initiated in the 30 days prior to death | 201 (4.9) | 16 (5.0) | 12 (4.6) | 0.961 |
| Any chemotherapy in the 14 days prior to death | 292 (7.2) | 20 (6.2) | 15 (5.7) | 0.550 |
| Patient died in hospital | 737 (18.1) | 80 (24.8) | 68 (25.8) | **<0.001** |
| Inpatient admission in the 30 days prior to death | 1,706 (42.0) | 173 (53.7) | 115 (43.7) | **<0.001** |
| ICU stay in the 30 days prior to death | 605 (14.9) | >55 | <11 | **0.016** |
| Two or more ER visits in the 30 days prior to death | 104 (2.6) | >11 | <11 | 0.210 |
| No hospice use prior to death | 1,223 (30.1) | 115 (35.7) | 98 (37.3) | **0.008** |
| Late initiation of hospice (in the 3 days prior to death) | 371 (9.1) | 32 (9.9) | 19 (7.2) | 0.496 |
