# Supplementary material for: Health Care Access Dimensions and Racial Disparities in End-of-Life Care Quality among Patients with Ovarian Cancer
Source: Cancer Res Commun. 2024 Mar 18;4(3):811–21. doi: 10.1158/2767-9764.CRC-23-0283 (PMC10946308; doi:10.1158/2767-9764.CRC-23-0283)
Supplement: Supplementary Table 4 — Relative risk ratio estimates of the associations between HCA dimensions, patient race/ethnicity, and EOL care quality outcomes [file crc-23-0283-s05.docx]

| **Supplementary Table 4:** Relative risk ratio estimates of the associations between HCA dimensions, patient race/ethnicity, and EOL care quality outcomes (N=4,646) | | | | |
| --- | --- | --- | --- | --- |
|  | ***Patient characteristics + affordability score**** | ***Patient characteristics + availability score**** | ***Patient characteristics + accessibility score**** | ***Patient characteristics +***  ***3 HCA scores**** |
| **Died in the hospital** |  |  |  |  |
| Affordability Score | **0.91 (0.85-0.98)** |  |  | **0.91(0.84-0.98)** |
| Availability Score |  | **1.09 (1.01-1.19)** |  | **1.11(1.02-1.21)** |
| Accessibility Score |  |  | **0.89 (0.79-1.00)** | 0.91(0.81-1.03) |
| Race (ref = NHW) |  |  |  |  |
| NHB | **1.26 (1.02-1.56)** | **1.34 (1.09-1.64)** | 1.28 (1.05-1.56) | **1.27(1.03-1.57)** |
| Hispanic | 1.20 (0.96-1.50) | **1.30 (1.05-1.62)** | 1.20 (0.97-1.48) | 1.23(0.98-1.54) |
| **Hospitalized in last 30 days of life** |  |  |  |  |
| Affordability Score | 1.00 (0.96-1.04) |  |  | 0.99 (0.95-1.03) |
| Availability Score |  | **1.07 (1.02-1.12)** |  | **1.07 (1.02-1.12)** |
| Accessibility Score |  |  | 1.04 (0.98-1.11) | 1.04 (0.96-1.11) |
| Race (ref = NHW) |  |  |  |  |
| NHB | **1.18 (1.05-1.32)** | **1.17 (1.05-1.31)** | **1.13 (1.02-1.26)** | **1.16 (1.03-1.30)** |
| Hispanic | 1.02 (0.88-1.17) | 1.02 (0.89-1.18) | 0.97(0.84-1.11) | 1.01 (0.88-1.17) |
| **Intensive Care Unit stay in last 30 days of life** |  |  |  |  |
| Affordability Score | 0.94 (0.87-1.02) |  |  | **0.90 (0.83-0.98)** |
| Availability Score |  | 1.01 (0.92-1.11) |  | 1.04 (0.95-1.15) |
| Accessibility Score |  |  | **1.29 (1.01-1.52)** | **1.35 (1.14-1.60)** |
| Race (ref = NHW) |  |  |  |  |
| NHB | 1.19(0.93-1.52) | 1.23 (0.97-1.57) | 1.14(0.89-1.45) | 1.10 (0.86-1.41) |
| Hispanic | 1.14(0.88-1.49) | 1.19 (0.92-1.54) | 1.12(0.87-1.45) | 1.09 (0.83-1.42) |
| **Did not initiate hospice prior to death** |  |  |  |  |
| Affordability Score | 0.95 (0.91-1.00) |  |  | 0.97 (0.92-1.02) |
| Availability Score |  | 1.05 (0.99-1.11) |  | 1.06 (1.00-1.12) |
| Accessibility Score |  |  | **0.86 (0.79-0.93)** | **0.87 (0.80-0.95)** |
| Race (ref = NHW) |  |  |  |  |
| NHB | **1.20 (1.02-1.40)** | **1.23 (1.05-1.44)** | **1.27 (1.09-1.48**) | **1.23 (1.04-1.44)** |
| Hispanic | 1.09 (0.92-1.29) | 1.13 (0.96-1.33) | 1.15 (0.98-1.35) | 1.11 (0.94-1.32) |
| **Initiated new chemotherapy agent within 30 days of death** |  |  |  |  |
| Affordability Score | 1.17(1-1.37) |  |  | 1.19 (1.00-1.40) |
| Availability Score |  | 0.99 (0.82-1.20) |  | 0.95 (0.78-1.16) |
| Accessibility Score |  |  | 1.06 (0.80-1.40) | 0.96 (0.72-1.30) |
| Race (ref = NHW) |  |  |  |  |
| NHB | 1.09 (0.63-1.88) | 0.99(0.58-1.69) | 0.98 (0.57-1.68) | 1.10 (0.64-1.91) |
| Hispanic | 0.98 (0.53-1.82) | 0.88(0.48-1.62) | 0.88 (0.48-1.62) | 0.98 (0.53-1.83) |
| **Received chemotherapy within 14 days of death** |  |  |  |  |
| Affordability Score | 1.02 (0.89-1.16) |  |  | 1.03 (0.89-1.19) |
| Availability Score |  | 1.07 (0.92-1.26) |  | 1.07 (0.91-1.25) |
| Accessibility Score |  |  | 0.89 (0.71-1.10) | 0.87 (0.70-1.09) |
| Race (ref = NHW) |  |  |  |  |
| NHB | 0.83 (0.51-1.35) | 0.82(0.51-1.32) | 0.84 (0.52-1.35) | 0.86 (0.52-1.39) |
| Hispanic | 0.79 (0.45-1.37) | 0.79(0.46-1.36) | 0.79 (0.46-1.36) | 0.81 (0.47-1.41) |
| **2 or more ER visits in last 30 days of life** |  |  |  |  |
| Affordability Score | **0.76 (0.61-0.96)** |  |  | 0.87(0.68-1.12) |
| Availability Score |  | 1.20 (0.94-1.54) |  | 1.23(0.96-1.58) |
| Accessibility Score |  |  | **0.44 (0.33-0.58)** | **0.46(0.35-0.62)** |
| Race (ref = NHW) |  |  |  |  |
| NHB | 1.24 (0.66-2.33) | 1.43 (0.77-2.64) | 1.75 (0.94-3.25) | 1.59 (0.84-3.01) |
| Hispanic | 0.56 (0.22-1.43) | 0.70 (0.28-1.76) | 0.74 (0.29-1.87) | 0.71 (0.28-1.82) |
| **Late initiation of hospice (within 3 days of death)** |  |  |  |  |
| Affordability Score | 1.10 (0.97-1.24) |  |  | 1.12 (0.99-1.27) |
| Availability Score |  | 0.89 (0.77-1.03) |  | 0.87 (0.74-1.01) |
| Accessibility Score |  |  | 1.07(0.87-1.33) | 1.02 (0.81-1.27) |
| Race (ref = NHW) |  |  |  |  |
| NHB | 1.13 (0.76-1.68) | 1.07 (0.72-1.59) | 1.05 (0.71-1.56) | 1.15 (0.77-1.72) |
| Hispanic | 0.75 (0.46-1.23) | 0.69 (0.42-1.13) | 0.70 (0.43-1.14) | 0.74 (0.45-1.22) |
| *Models additionally adjusted for age, tumor stage, tumor histology, patient comorbid conditions, geographic region of residence at diagnosis, and year of diagnosis. HCA: healthcare access; NHW: non-Hispanic White; NHB: non-Hispanic Black | | | | |
