# Supplementary material for: Health Care Access Dimensions and Racial Disparities in End-of-Life Care Quality among Patients with Ovarian Cancer
Source: Cancer Res Commun. 2024 Mar 18;4(3):811–21. doi: 10.1158/2767-9764.CRC-23-0283 (PMC10946308; doi:10.1158/2767-9764.CRC-23-0283)
Supplement: Supplementary Table 5 — Relative risk ratio (RR) for receipt of any chemotherapy within 14 days of death with interaction term between accessibility score and patient race [file crc-23-0283-s06.docx]

| **Supplemental Table 5**: Relative risk ratio (RR) for receipt of any chemotherapy within 14 days of death with interaction term between accessibility score and patient race (N=4,646) | |
| --- | --- |
| *Parameter* | *RR (95% CI)* |
| Accessibility Score*Patient Race (NHB) | 1.82 (0.41-8.16) |
| Accessibility Score*Patient Race (Hispanic) | **0.42 (0.21-0.81)** |
| Affordability Score | 1.02 (0.89-1.17) |
| Availability Score | 1.07 (0.92-1.24) |
| Accessibility Score | 0.91 (0.73-1.14) |
| Race (ref = NHW) |  |
| NHB | 0.79 (0.48-1.32) |
| Hispanic | 0.85 (0.50-1.44) |

*Model additionally adjusted for age, tumor stage, tumor histology, patient comorbid conditions, geographic region of residence at diagnosis, and year of diagnosis.

NHW: non-Hispanic White; NHB: non-Hispanic Black
